# Supplementary material for: Biased belief updating and suboptimal choice in foraging decisions
Source: Nat Commun. 2020 Jul 9;11:3417. doi: 10.1038/s41467-020-16964-5 (PMC7347922; doi:10.1038/s41467-020-16964-5)
Supplement: Supplementary file 1 — Supplementary Information [file 41467_2020_16964_MOESM1_ESM.pdf]

Supplementary Information

Biased belief updating and suboptimal choice in foraging decisions

Garrett et al.

Supplementary Notes

Block wise learning and order effects. In the main paper, to simplify the analysis, we collapsed the two intermediate options (LDLR and HDHR) into one intermediate category. The same pattern of results is found however if these are treated separately along with the other two options (LDHR and HDLR) (**Supplementary Fig. 1**). Specifically, a repeated measures ANOVA looking at the percentage of accept decisions with option (LDHR, LDLR, HDHR, HDLR) and environment (rich, poor) as repeated factors revealed a main effect of environment (Experiment 1: F(1,39) = 31.75, p<0.001, partial η^2 = 0.45; Experiment 2: F(1,37) = 16.44, p<0.001, partial η^2 = 0.31; Experiment 3: F(1, 37) = 29.63, p<0.001 partial η^2 = 0.45), a main effect of option (Experiment 1: F(3,117) = 154.17, p<0.001, partial η^2 = 0.80; Experiment 2: F(3, 111) = 133.65, p<0.001, partial η^2 = 0.78; Experiment 3: F(3, 111) = 117.82, p<0.001, partial η^2 = 0.76) and an environment by option interaction (Experiment 1: F(3,117) = 26.47, p<0.001, partial η^2 = 0.40; Experiment 2: F(3,35) = 25.25, p<0.001, partial η^2 = 0.41; Experiment 3: F(3,35) = 32.24, p<0.001, partial η^2 = 0.47).

To separately examine differences in blockwise learning between participants we ran the same ANOVA with option (LDHR, LDLR, HDHR, HDLR) and environment (rich, poor) as repeated factors but now also included order condition (RichPoor, PoorRich) as a between subjects' factor. As reported in the main text (where the intermediate options are collapsed), this revealed an interaction between environment and order condition in Experiment 1 (F(1,38) = 11.64, p = 0.002, partial η^2 = 0.23) and Experiment 2 (F(1,36) = 4.33, p = 0.045, partial η^2 = 0.11). As expected, this interaction was not significant in Experiment 3 (F(1,36) = 0.57, p=0.45, partial η^2 = 0.02).

Financial cost. To quantify the cost of the learning asymmetry we observed, compared to a model without bias, we ran a separate set of simulations under asymmetric and symmetric learning (using the average learning rates from the model fitting procedure) and calculated average earnings under each (see **Methods**). This revealed that symmetric learners earned 10% more than asymmetric learners on average over the course of the experiment (t(78) = 10.03, p<0.001, 95% CI [10.84, 16.21], two tailed independent sample ttest comparing earnings under symmetric learning versus asymmetric learning).

Preference between intermediate options. The two intermediate options, LDLR and HDHR, were equated in terms of the reward they provided per second and were encountered with equal frequency in each environment (**Fig. 1c**). Although the core idea of reward rate maximization and the MVT seem to predict equivalence between two options with the same reward rate, participants tended to accept one of the two intermediate options (small rewards quickly) more than another (larger rewards slowly), despite these having equal reward rate (Experiment 1: Mean Difference in Acceptance rates = 24%, t(39) = 3.95, p<0.001, 95% CI [0.12, 0.36]; Experiment 2: Difference in Acceptance rates = 12%, t(37) = 2.20, p=0.034, 95% CI [0.01, 0.24], two tailed paired sample ttest on acceptance rates for LDLR versus HDHR averaged across both environments. See also **Supplementary Fig. 1**). This preference did not replicate significantly in the third experiment but the pattern was in the same direction (Experiment 3: t(37) = 1.27, p=0.21, 95% CI [-0.04, 0.17]).

In fact, this asymmetry is also predicted by the model (Experiment 1: Difference in Acceptance rates = 22%, t(999) = 125.35, p<0.001; Experiment 2: Difference in Acceptance

rates = 15%; t(999) = 93.87, p<0.001, two tailed paired sample ttest comparing LDLR versus HDHR acceptance rates, averaged over both environments from the Asymmetric Model simulations) and constrains the form of its choice rule. In particular, the choice rule is expressed in terms of reward amount (the difference between the reward on offer and the opportunity cost, in units of reward, for occupying that time) rather than the alternative of comparing these quantities expressed as rates (normalized by delay). Softmax choice on the former basis results in a larger decision variable and more deterministic choices for the longer-delay option; if the net decision variable is negative (typically the case in our regime when $\alpha^+ > \alpha^-$, because the opportunity cost is overestimated), the shorter one will be rejected less often. Other features not included in the model, such as time discounting, might also contribute to this preference.

Perseverance Models. To examine the possibility that the order effect we observe in Experiment 1 and in Experiment 2 could be accounted for by choice perseveration as opposed to learning asymmetry (following[1]) we fitted a model with a perseveration parameter (β_{stick}) as an additional free parameter to participant choices in the first two experiments. In these models, the softmax was formulated as:

$$P(accept) = \frac{exp(\beta_1 * r_i + \beta_{stick} * I(c_{t-1} = accept))}{exp(\beta_1 * r_i + \beta_{stick} * I(c_{t-1} = accept)) + exp(\beta_0 + \beta_1 * c_i + \beta_{stick} * I(c_{t-1} = reject))}$$

$I(c_{t-1} = accept)$ and $I(c_{t-1} = reject)$ are binary indicators, indicating whether the choice on the previous trial was to accept or reject respectively. If β_{stick} is positive (negative) therefore, the value of the previous trials choice is increased (decreased). All other aspects of the model were exactly as described for the Symmetric Model (see **Methods**), with a single learning rate (α) used to update the environments rate of reward. Comparing Leave One Out Cross Validation scores between the two models (via two tailed paired sample ttests) revealed that the Asymmetric Model again provided a superior fit to choices compared to the Perseverance Model (Experiment 1: t(39) = 7.84, p<0.001, 95% CI [19.92, 33.88]; Experiment 2: t(37) = 7.79, p<0.001, 95% CI [12.38, 21.09]). There was also no significant improvement in scores between the Perseverance Model and the Symmetric Model (Experiment 1: t(39) = 0.59, p=0.56, 95% CI [-1.99, 3.62]; Experiment 2: t(37) = 1.62, p=0.11, 95% CI [-0.66, 5.89]). Simulations revealed that the perseverance model was also not able to qualitatively capture the order effect we observed (see **Supplementary Fig. 4)**

Learn Options Models. The Symmetric Model and Asymmetric Model described in the main text assume that the rewards and time investment (r_i and t_i) of each of the 4 options (i = {1,2,3,4}) were known from the outset. This seems a plausible assumption since the options were visually very distinct, outcomes (rewards and delays) easily observable and were stationary (i.e. each option always provided the exact same r_i, and t_i). Nonetheless, it may be the case that individuals learnt the rewards and time investments associated with each option over time following feedback. To test whether this was the case, we augmented the Symmetric Model and Asymmetric Model so that the reward and time investment of each option was learnt. Specifically, we initialized a set of reward (Qr_i) and time (Qt_i) Q values for each option to 0 (where i indexes the option from 1 to 4). Each of these Q values was then updated following acceptance of an option according to two delta rules (one for Qr and one for Qt) as follows:

(1) $Qr_{i,t+1} = Qr_{i,t} + \lambda \ \delta_t$

(2) $Qt_{i,t+1} = Qt_{i,t} + \lambda \ \delta_t$

δ is a prediction error, calculated for reward and time respectively as:

(3) $\delta_t = r_i - \mathrm{Qr}_{i,t}$

(4) $\delta_t = t_i - \mathrm{Qt}_{i,t}$

where r is the reward received and t is the time investment required following acceptance of an option. λ is the learning rate used to update estimates of the reward and time investment associated with each option (we use λ rather than α to distinguish it from the learning rate used to update estimates of the environments reward rate).

The opportunity cost (c_i), rather than being the product of the actual time investment required and the estimated reward rate (per second) of the environment (ρ) is now calculated as the current estimate of the time that the option takes to pursue (Qt_i) multiplied by the estimated reward rate (per second) of the environment (ρ):

(5) $c_{i,t} = \rho_t \mathrm{Qt}_{i,t}$

The decision to accept or reject is now calculated as the difference between the estimated opportunity cost ($c_{i,t}$) and the current estimate of the reward that the option will gain (Qr_i). As before, this was implemented in a softmax function:

(6) $$P(accept) = \frac{1}{1+exp\left(\beta_0 - \beta_1\left(\mathrm{Qr}_{i,\ t} - c_{i,\ t}\right)\right)}$$

We implemented this extra learning component for both the Symmetric and the Asymmetric Model (for each experiment) with everything else exactly as described for these models. In each case we used a different learning rate (λ) to model learning of the rewards/costs associated with each option to the learning rate(s) used to model learning of the environments rate of reward (α in the case of the Symmetric Model, α^+ and α^- in the case of the Asymmetric Model).

In each experiment, as we found previously (when r_i and t_i were assumed to be correctly known from the outset rather than learnt following feedback), a model with two learning rates for the environments rate of reward (Asymmetric Learn Options Model) provided a better fit to the data compared to a model where this rate was updated using a single learning rate (Symmetric Learn Options Model). The asymmetry between α^+ and α^- was again biased in a positive direction in each experiment (Experiment 1: z = 2.91, p<0.01; Experiment 2: z = 3.10, p<0.01; Experiment 3: z = 2.73, p<0.01, **Supplementary Table 1**).

Supplementary Tables

Supplementary Table 1: model fitting and parameters for the Learn Option models for each experiment

Experiment / Model	LOOCV	λ	α	α^+	α^-	β_0	β_1
Experiment 1 (N=40)							
Symmetric Learn Options Model	93.93 (3.33)	0.4072 95% CI = [0.2789, 0.5463]	0.0177 95% CI = [0.005, 0.0515]	-	-	0.86 95% CI = [0.59, 1.13]	0.09 95% CI = [0.08, 0.10]
Asymmetric Learn Options Model	67.94*** (4.04)	0.5489 95% CI = [0.3864, 0.7035]	-	0.0066 95% CI = [0.0035, 0.0118]	0.0020 95% CI = [0.0011, 0.0037]	-1.82 95% CI = [-2.54, -1.11]	0.09 95% CI = [0.08,0.11]
Experiment 2 (N=38)							

Symmetric Learn Options Model	62.28 (2.68)	0.2390<br>95% CI = [0.1762, 0.3123]	0.0213<br>95% CI = [0.007, 0.056]	-	-	0.69<br>95% CI = [0.40, 0.98]	0.10<br>95% CI = [0.09, 0.12]
Asymmetric Learn Options Model	47.92*** (2.82)	0.3076<br>95% CI = [0.2213, 0.4062]	-	0.0051<br>95% CI = [0.0031, 0.0082]	0.0022<br>95% CI = [0.0014, 0.0034]	-1.37<br>95% CI = [-2.00, -0.74]	0.11<br>95% CI = [-2.00, -0.74]
Experiment 3 (N=38)							
Symmetric Learn Options Model	77.67 (3.53)	0.3332<br>95% CI = [0.2149, 0.4710]	0.1116<br>95% CI = [0.0491, 0.2171]	-	-	0.65<br>95% CI = [0.38, 0.93]	0.11<br>95% CI = [0.09, 0.13]
Asymmetric Learn Options Model	65.79*** (3.39)	0.3285<br>95% CI = [0.2057, 0.4733]	-	0.0293<br>95% CI = [0.0218, 0.0389]	0.0179<br>95% CI = [0.0139, 0.0227]	-1.01<br>95% CI = [-1.44, -0.57]	0.11<br>95% CI = [0.09, 0.13]

Supplementary Table 1: model fitting and parameters across the three experiments for models which incorporate learning of the rewards and time investment associated with each option. The table summarizes for each model its fitting performances and its average parameters: LOOCV: mean (standard error of the mean) leave one out cross validation scores over participants; α: learning rate for both positive and negative prediction errors (Symmetric Learn Options Model); $\alpha+$: learning rate for positive prediction errors; $\alpha-$: average learning rate for negative prediction errors (Asymmetric Learn Options Model); λ: learning rate for rewards and time investment associated with each option; β_0: softmax intercept (bias towards reject); β_1: softmax slope (sensitivity to the difference in the value of rejecting versus the value of accepting an option). Data for model parameters are expressed as mean and 95% confidence intervals (calculated as the sample mean +/- 1.96*standard error).
***P<0.001 comparing LOOcv scores between Symmetric Learn Options Model and the Asymmetric Learn Options Model, two sided paired sample ttest (Experiment 1: t(39) = 7.24, p = 9.9395E-9; Experiment 2: t(37) = 6.10, p = 4.6148E-7; Experiment 3: t(37) = 5.61, p = 0.000002)
Source data are provided as a Source Data file.

Supplementary Figures

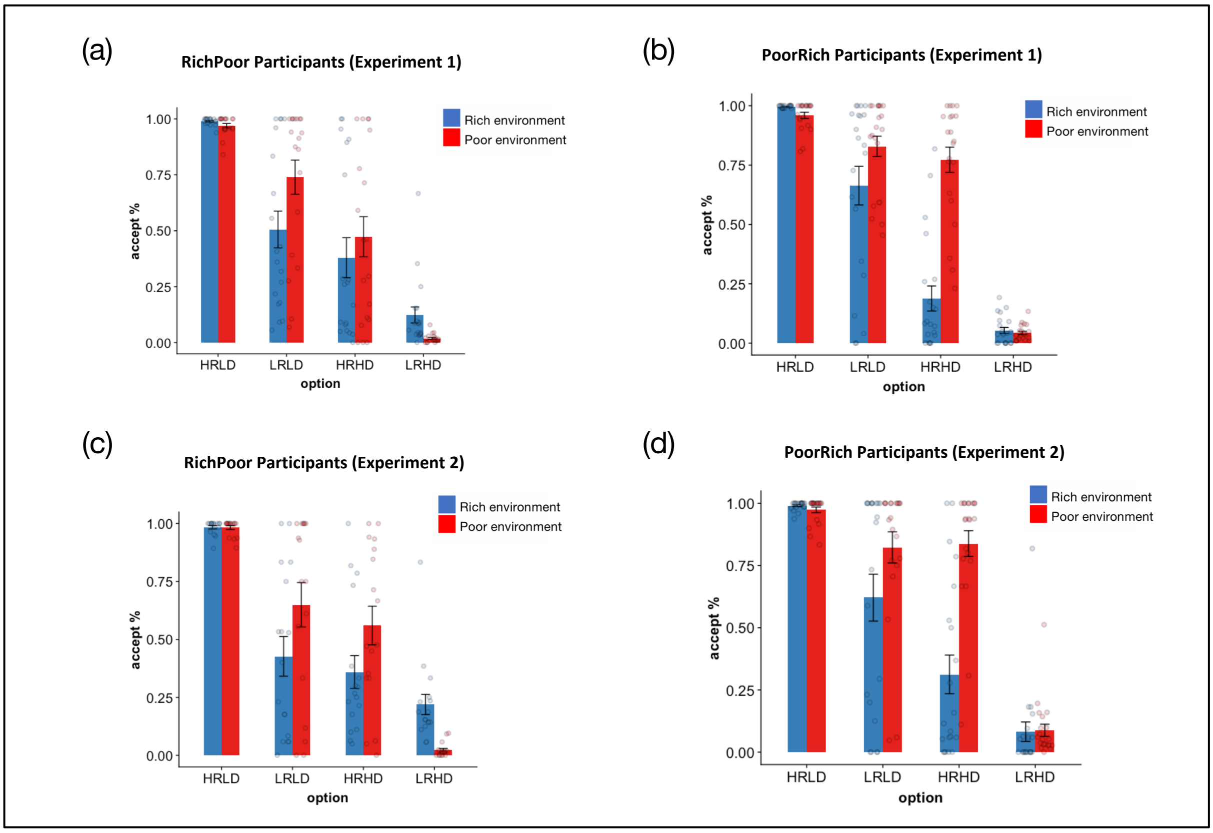

Supplementary Figure 1.
Acceptance rates (%) for each option in each environment separately for each group of participants (a) Experiment 1 RichPoor group (N=19), (b) Experiment 1, PoorRich group (N=21), (c) Experiment 2 RichPoor group (N=17), (d) Experiment 2 PoorRich group. As reported in the main text, there was an interaction between environment and order condition in Experiment 1 ($F(1,38) = 11.64$, $p = 0.002$, partial $\eta^2 = 0.23$, Repeated measures ANOVA with option [LDHR, LDLR, HDHR, HDLR] and environment [rich, poor] as factors) and Experiment 2 ($F(1,36) = 4.33$, $p = 0.045$, partial $\eta^2 = 0.11$ Repeated measures ANOVA with option [LDHR, LDLR, HDHR, HDLR] and environment [rich, poor] as factors). LDHR = low delay, high reward option; LDLR = low delay, low reward option; HDHR = high delay, high reward option; HDLR = high delay, low reward option
Dots represent individual data points, bars represent the group mean. Error bars represent mean +/- standard error of the mean.
Source data are provided as a Source Data file.

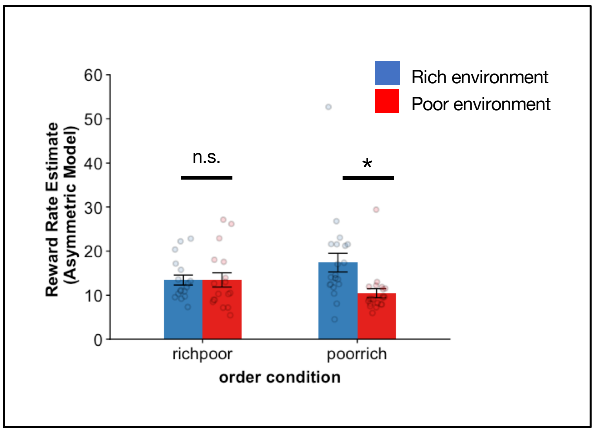

Supplementary Figure 2.
Consistent with Experiment 1 (main text and Figure 4a), extracting reward rate estimates (ρ) from the Asymmetric Model for each participant in each experimental block in Experiment 2 (PoorRich N=21, RichPoor N=17) revealed a significant environment by condition interaction ($F(1, 36) = 21.42$, $p = 0.000046$, partial $\eta^2 = 0.37$). This arose out of a significant difference in ρ between environments for participants in the PoorRich condition ($t(20) = 6.08$, p =

0.000006, 95% CI [4.56, 9.32], two tailed paired sample ttest) which was absent among participants assigned to the RichPoor condition (t(16) = 0.004, 95% CI [-1.87, 1.88], p = 0.997).
*p<0.05, paired sample ttest
n.s. = non significant (p>0.05)
Dots represent individual data points, bars represent the group mean. Error bars represent mean +/- standard error of the mean.
Source data are provided as a Source Data file.

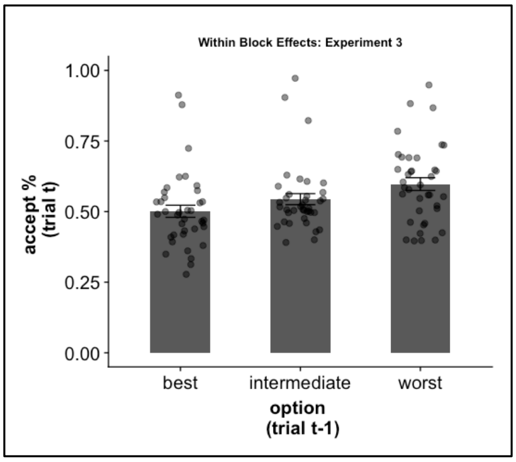

Supplementary Figure 3.
As observed in Experiments 1 and 2 (see main text and Figure 2), in Experiment 3 (N=38) acceptance rates were modulated by trial to trial dynamics. Repeated measures ANOVA with previous option (best, intermediate, worst) and environment (rich, poor) as factors (main effect of previous option: F(2, 74) = 21.02, p = 5.8932E-8, partial η = 0.36)
Dots represent individual data points, bars represent the group mean. Error bars represent mean +/- standard error of the mean.
Source data are provided as a Source Data file.

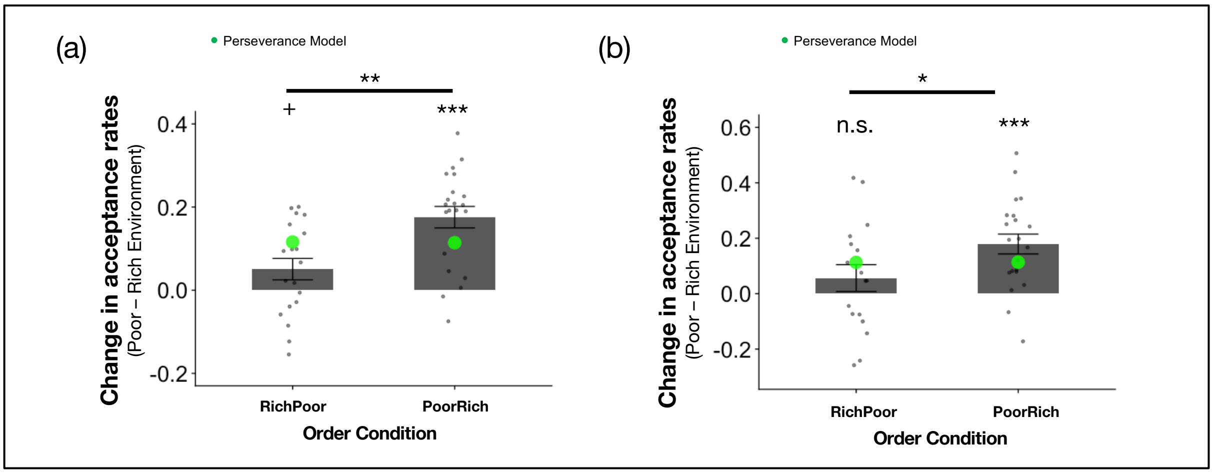

Supplementary Figure 4.
The Perseverance Model was able unable to recapitulate the order effect observed in (a) Experiment 1 (PoorRich N=21 independent participants, RichPoor N=19 independent participants) and (b) Experiment 2 (PoorRich N=21, RichPoor N=17). Grey dots represent individual data points, grey bars represent the group mean. Green circles represent the pattern of choices generated by simulations from the Perseverance Model. Error bars represent mean +/- standard error of the mean.
$^{+}$0.05<p<0.10; *p<0.05; ** p<0.01; *** p<0.001: independent sample ttest / paired sample ttest / one sample ttest (vs 0) as appropriate (all two sided); *n.s.* = non significant
Source data are provided as a Source Data file.

Supplementary References

1. Katahira, K. The statistical structures of reinforcement learning with asymmetric value updates. *J. Math. Psychol.* **87**, 31–45 (2018).
