# Supplementary material for: Biased belief updating and suboptimal choice in foraging decisions
Source: Nat Commun. 2020 Jul 9;11:3417. doi: 10.1038/s41467-020-16964-5 (PMC7347922; doi:10.1038/s41467-020-16964-5)
Supplement: Supplementary file 3 — Reporting Summary [file 41467_2020_16964_MOESM3_ESM.pdf]

nature research

Corresponding author(s): Neil Garrett

Last updated by author(s): May 12, 2020

Reporting Summary

Nature Research wishes to improve the reproducibility of the work that we publish. This form provides structure for consistency and transparency in reporting. For further information on Nature Research policies, see Authors & Referees and the Editorial Policy Checklist.

Statistics

For all statistical analyses, confirm that the following items are present in the figure legend, table legend, main text, or Methods section.

n/a	Confirmed	
☐	☒	The exact sample size (n) for each experimental group/condition, given as a discrete number and unit of measurement
☐	☒	A statement on whether measurements were taken from distinct samples or whether the same sample was measured repeatedly
☐	☒	The statistical test(s) used AND whether they are one- or two-sided<br>*Only common tests should be described solely by name; describe more complex techniques in the Methods section.*
☐	☒	A description of all covariates tested
☒	☐	A description of any assumptions or corrections, such as tests of normality and adjustment for multiple comparisons
☐	☒	A full description of the statistical parameters including central tendency (e.g. means) or other basic estimates (e.g. regression coefficient) AND variation (e.g. standard deviation) or associated estimates of uncertainty (e.g. confidence intervals)
☐	☒	For null hypothesis testing, the test statistic (e.g. F, t, r) with confidence intervals, effect sizes, degrees of freedom and P value noted<br>*Give P values as exact values whenever suitable.*
☒	☐	For Bayesian analysis, information on the choice of priors and Markov chain Monte Carlo settings
☒	☐	For hierarchical and complex designs, identification of the appropriate level for tests and full reporting of outcomes
☐	☒	Estimates of effect sizes (e.g. Cohen's d, Pearson's r), indicating how they were calculated

Our web collection on statistics for biologists contains articles on many of the points above.

Software and code

Policy information about availability of computer code

Data collection	Data collection was programmed in JavaScript using the toolbox jsPsych (Leeuw, 2015) version 5.0.3.
Data analysis	Data analysis was conducted using Rstudio and SPSS. Computational models were fit using Julia.

For manuscripts utilizing custom algorithms or software that are central to the research but not yet described in published literature, software must be made available to editors/reviewers. We strongly encourage code deposition in a community repository (e.g. GitHub). See the Nature Research guidelines for submitting code & software for further information.

Data

Policy information about availability of data

All manuscripts must include a data availability statement. This statement should provide the following information, where applicable:

- Accession codes, unique identifiers, or web links for publicly available datasets
- A list of figures that have associated raw data
- A description of any restrictions on data availability

All data and code has been made available publicly (via Github). The source data underlying all Figures and Tables are provided as a Source Data file.

Field-specific reporting

Please select the one below that is the best fit for your research. If you are not sure, read the appropriate sections before making your selection.

☐ Life sciences ☒ Behavioural & social sciences ☐ Ecological, evolutionary & environmental sciences

For a reference copy of the document with all sections, see nature.com/documents/nr-reporting-summary-flat.pdf

Behavioural & social sciences study design

All studies must disclose on these points even when the disclosure is negative.

Study description	Data are responses from participants.
Research sample	Mturk participants
Sampling strategy	n/a
Data collection	Participants were recruited online via Amazon Mechanical Turk. Participants completed the study via an Internet browser using their computer keyboard.
Timing	10/07/2017 (experiment 1); 06/05/2018 (experiment 2); 16/05/2018
Data exclusions	Following best practice for studies with this population (Crump et al., 2013), several a priori exclusion criteria were applied to ensure data quality. Participants were excluded if any of the following applied: (1) Did not finish the task (n=5); (2) Made 20 or more missed responses (n=14); (3) Made 10 or more incorrect force trial responses (either accepting options when forced to reject or rejecting options when forced to accept, n=1); (4) Poor discriminability, defined as choosing the worst option (low reward, high delay) a greater percentage of times than the best option (high reward, low delay) or accepting/rejecting all options on every single trial (n=2).
Non-participation	n/a
Randomization	Participants were randomly allocated to RichPoor or PoorCondition. This was determined by a random number generator in the program used to collect the data.

Reporting for specific materials, systems and methods

We require information from authors about some types of materials, experimental systems and methods used in many studies. Here, indicate whether each material, system or method listed is relevant to your study. If you are not sure if a list item applies to your research, read the appropriate section before selecting a response.

Materials & experimental systems

n/a	Involved in the study	
☒	☐	Antibodies
☒	☐	Eukaryotic cell lines
☒	☐	Palaeontology
☒	☐	Animals and other organisms
☐	☒	Human research participants
☒	☐	Clinical data

Methods

n/a	Involved in the study	
☒	☐	ChIP-seq
☒	☐	Flow cytometry
☒	☐	MRI-based neuroimaging

Human research participants

Policy information about studies involving human research participants

Population characteristics	Experiment 1: mean ± standard deviation (s.d.) age: 32.73 (7.94); 11/40 participants female. All US residents.<br>Experiment 2: mean ± standard deviation (s.d.) age: 33.71 (8.83); 16/38 participants female. All US residents.<br>Experiment 3: mean ± standard deviation (s.d.) age: 34.63 (8.58); 12/38 participants female. All US residents.
Recruitment	Participants were recruited online via Amazon Mechanical Turk.
Ethics oversight	Princeton University Institutional Review Board

Note that full information on the approval of the study protocol must also be provided in the manuscript.
